# National and rural-urban prevalence and determinants of early initiation of breastfeeding in India

**DOI:** 10.1186/s12889-019-7246-7

**Published:** 2019-07-08

**Authors:** Praween Senanayake, Elizabeth O’Connor, Felix Akpojene Ogbo

**Affiliations:** 1 Translational Health Research Institute (THRI), School of Medicine, Western Sydney University, Campbelltown Campus, Locked Bag 1797, Penrith, NSW 2571 Australia; 2 Medical Education Unit, School of Medicine, Western Sydney University, Locked Bag 1797, Penrith, NSW 2571 Australia; 3 Prescot Specialist Medical Centre, Welfare Quarters, Makurdi, Benue State Nigeria

**Keywords:** Early initiation, Breastfeeding, Children, India, Rural, Urban

## Abstract

**Background:**

Early initiation of breastfeeding (EIBF) reduces the risk of neonatal mortality. Previous studies from India have documented some factors associated with EIBF. However, those studies used data with limited sample size that potentially affect the application of the evidence. Additionally, the effectiveness of national breastfeeding programmes requires up-to-date analysis of new and robust EIBF data. The present study aimed to investigate the prevalence and determinants of EIBF in India and determine to what extent these factors differ by a mother’s residence in the rural or urban area.

**Methods:**

This study used information from a total weighted sample of 94,401 mothers from the 2015–2016 India National Family Health Survey. Multivariate logistic regression was used to investigate the association between the study factors and EIBF in India and rural-urban populations, after adjusting for confounders and sampling weight.

**Results:**

Our analysis indicated that 41.5% (95% confidence interval (CI): 40.9–42.5, *P* < 0.001) of Indian mothers initiated breastfeeding within 1-h post-birth, with similar but significant different proportions estimated for those who resided in rural (41.0, 95% CI: 40.3–41.6, *P* < 0.001) and urban (42.9, 95% CI: 41.7–44.2, P < 0.001) areas. Mothers who had frequent health service contacts and those with higher educational attainment reported higher EIBF practice. Multivariate analyses revealed that higher educational achievement, frequent antenatal care visits and birthing in a health facility were associated with EIBF in India and rural populations (only health facility birthing for urban mothers). Similarly, residing in the North-Eastern, Southern, Eastern and Western regions were also associated with EIBF. Birthing through caesarean, receiving delivery assistance from non-health professionals and residing in rural areas of the Central region were associated with delayed EIBF in all populations.

**Conclusion:**

We estimated that more than half of Indian mothers delayed breastfeeding initiation, with different rural-urban prevalence. Key modifiable factors (higher maternal education and frequent health service contacts) were associated with EIBF in India, with notable difference in rural-urban populations. Our study suggests that targeted and well-coordinated infant feeding policies and interventions will improve EIBF for all Indian mothers.

**Electronic supplementary material:**

The online version of this article (10.1186/s12889-019-7246-7) contains supplementary material, which is available to authorized users.

## Background

Early initiation of breastfeeding (EIBF, defined as the provision of only breast milk to the newborn within the first hour of birth) has been well-documented to reduce the risk of neonatal mortality [1–4]. The protective effect of EIBF is based on the immunological components of the breast milk [5, 6], the improvement in exclusive breastfeeding [7–9] and the avoidance of prelacteal foods that deprive newborns of colostrum, rich in nutrients and immunoglobulins needed to fight disease [10, 11]. Despite the evidence supporting the protective effect of EIBF, the prevalence of EIBF remains low (an average of 50%) in many developing countries [12, 13], where the majority of preventable neonatal deaths exist [6], compared to the global recommendation of 90% [14]. Although India has made some progress in increasing EIBF rates in the past decade [15], evidence from regional areas indicated that the proportion of mothers who put their babies to the breast within the first hour of birth remains below the expected level, ranging from 36% [16] to 42% [17, 18].

A previous national study based on the 2005–2006 India National Family Health Survey (NFHS-3) [19] has elucidated factors associated with delayed EIBF in India. These attributes included caesarean delivery and living in the Central region. In contrast, health facility birthing, listening to the radio, frequent antenatal visits and living in the North-eastern, Southern or Western region were associated with increased likelihood of EBIF in India [19]. However, findings from these studies may not provide a current evidence base on EIBF in India. Similarly, it is unclear whether EIBF behaviour has changed in the past decade because of the implementation of a number of maternal and child health (MCH) interventions (e.g. Reproductive, Maternal, Newborn, Child, and Adolescent Health [RMNCH+A] Strategy [20], National Rural Health Mission, NRHM [21] and National Urban Health Mission under the National Health Mission [22]) and sample size differences.

In the NFHS-3, approximately 110,000 households were selected nationally from 1 billion people based on the 2001 census frame [23] compared to nearly 572,000 selected households from 1.2 billion people based on the 2011 census list in the 2015–2016 India National Family Health Survey (NFHS-4), [24, 25], which is the data source for the present study. Notably, the NFHS-4 has been documented to serve as the benchmark for future national household surveys in India [24–26]. The availability of new and more nationally representative data calls for up-to-date evidence to inform EIBF programmes and help policy decision-makers and health administrators provide targeted breastfeeding policies and interventions.

Additionally, the past nationwide study from India did not examine the differences in the factors associated with EIBF in both rural and urban populations of India given the implementation of both rural- and urban-specific MCH interventions [21, 22]. Reports have indicated that there are significant disparities in socioeconomic status and health service access in India, with subsequent impact on disease burden and health status [27–30], and whether these factors have an impact on EIBF remains unclear. An investigation into the factors associated with EIBF across rural-urban residence is crucial to understand where additional intervention is needed to meet the national and subnational breastfeeding targets. The present study aimed to investigate the prevalence and determinants of EIBF at the national level in India, along with those for rural-urban populations.

## Methods

### Data sources

The NFHS-4 data, also known as the India Demographic and Health Survey (DHS) were used for this study, collected by the International Institute for Population Sciences (IIPS), Mumbai through the Ministry of Health and Family Welfare (MoHFW), Government of India. The Inner City Fund (ICF) International, Maryland, USA provided technical assistance in data collection efforts. Infant and young child feeding practices (including EIBF) data, as well as socio-demographic and household characteristics were collected from a nationwide representative sample of women aged 15–49 years. The response rates in the interview varied across the states and territories of India, from 94.0% in Andhra Pradesh and West Bengal [24, 25] to 99.6% in Bihar [26].

Using a two-stage sampling design, a total sample of approximately 572,000 households (including women aged 15–49 years and men aged 15–54 years) across both rural and urban areas was obtained for the NFHS-4, with villages and census enumeration blocks as the primary sampling units, respectively. The NFHS-4 was based on the 2011 census, where an urban area constitutes statutory towns, census towns and outgrowths, while all areas other than urban were rural. The basic unit for rural areas is the revenue village. Specific exposition of what statutory towns, census towns and outgrowths mean are provided elsewhere [31].To obtain the sample of mothers who initiated breastfeeding within the first hour of birth, we restricted our analyses to the youngest living children aged less than 24 months, living with respondent (women aged 15–49 years) to reduce the potential effect of recall bias [32]. The total weighted sample was 94,104 for the total population of India, 68,260 for rural areas and 25,843 for urban areas. Additional information on the survey methodology is provided in the final India DHS reports [24, 25].

### Outcome variable

In the present study, early or timely initiation of breastfeeding was measured as the proportion of infants 0–23 months of age who were put to the breast within the first hour of birth, in line with the World Health Organisation and United Nations Children’s Funds (WHO/UNICEF) definitions for assessing infant and young child feeding practices [33].

### Study variables

We selected study factors based on the evidence from previous studies [34–36], including socio-economic, individual and health service factors. Socio-economic factors included the mother’s highest educational level and employment status, household wealth index and father’s highest educational level. The household wealth index was derived from a principal components analysis conducted by the IIPS and ICF International and was calculated as a score of ownership household assets such as transportation device, ownership of durable goods and household facilities [37]. The household wealth index was classified into five categories (quintiles), and each household was assigned to one of these wealth index categories, namely: poorest, poorer, middle, rich and richest. We re-categorised the bottom 40% of households as poor households, the next 40% as the middle households and the top 20% as rich households to provide sufficient numbers in each category, consistent with previously published studies [9, 35, 38]. Individual factors included maternal age, the gender of the child, preceding birth interval and birth order of the child (the position of the child in the family).

Health service factors included the number of antenatal care (ANC) visits, the place of delivery, the mode of delivery and the type of delivery assistance. Delivery assistance received from non-health professionals (i.e., assistance from outside the health facility) was categorised as either traditional birth attendants (TBAs or Dai in India) or other non-health professionals. A traditional birth attendant is commonly a woman, who assists the mother during childbirth and who originally acquired those birthing skills by working with other traditional birth attendants or by delivering babies herself [39]. Other non-health professionals included relatives, friends, no one and others, while health professionals included doctors, auxiliary nurse midwives, nurses, midwives, and lady health visitors [40].

Given that India is a federal union that comprise 29 states and 7 union territories, with a total of 36 entities, we also considered geographical region in the analyses. The states and union territories are aggregated into six zonal councils (North, South, East, West, Central and North-Eastern) to facilitate inter-state cooperation with regard to better socio-political and economic collaboration, and health system strengthening [19, 20].

### Statistical analysis

Our preliminary analyses involved the calculation of the frequencies (and percentages) of the study factors for the overall population and rural-urban residence, prevalence of EIBF in India and rural-urban residence, as well as a series of frequencies and cross-tabulations to estimate the prevalence of EIBF by the study factors for all three locations (overall population and rural-urban residence). This was followed by univariate logistic regression analysis to examine factors associated with EIBF in India and rural-urban residence. We only entered into the multivariate models, those study variables with *P*-value < 0.05 in univariate models to estimate the factors associated with EIBF in India and rural-urban residence. Univariate and multivariate odds ratios (ORs) and their 95% confidence intervals were reported in the present study for all three locations. All analyses were performed using the ‘svy’ command for calculation of counts and percentages in Stata 15.0 (Stata Corporation, College Station, Texas, USA) to adjust for sampling weight, clustering and stratification.

### Ethics

The Ethics Review Board at the International Institute for Population Sciences, Mumbai, India granted the DHS project ethical approvals before the surveys were conducted, with written informed consent obtained from participants during the surveys. Approval was sought from Measure DHS and permission was granted for this use.

## Results

### Characteristics of the study population

In the total weighted sample of 94,104 women aged 15–49 years, 59.3% had secondary and higher education in the total population, 13.5% had primary education and 27.3% had no education. In rural areas, a little above half (53.4%) of mothers had secondary and higher education, while 14.6% had primary education and 32.0% had no education. In contrast, the majority (74.6%) of mothers had secondary and higher education in the urban population, while 10.6% had primary education and 14.8% had no education [Additional file 1].

### Prevalence of EIBF in the study population

The proportion of mothers who initiated breastfeeding within the first hour of birth for children aged 0–23 months was 41.5% [95% confidence interval (CI): 40.9–42.5, *P* < 0.001] in the total population **[**Table 1**]**. The study found a higher proportion of mothers with secondary and above education level who put their babies to the breast within the first hour of birth in the total population [62.9% (95% CI: 62.1–63.8), *P* < 0.001]. Mothers who received delivery assistance from health professionals also reported a higher proportion of EIBF compared to those who were assisted by TBAs or other non-professionals in India. The prevalence of early initiation of breastfeeding was 41.0% (95% CI: 40.3–41.6, P < 0.001) in the rural population and 42.9% (95% CI: 41.7–44.2, *P* < 0.001) in the urban population **[**Table 1**],** reflecting a significant difference between EIBF prevalence among mothers who resided in rural areas compared to those who lived in urban areas of India. There was a higher proportion of EIBF mothers with secondary and above education level in the urban population (76.8, 95% CI: 75.1–78.5%, *P* < 0.001) compared to the rural population (57.4, 95% CI: 56.5–58.4%, P < 0.001).

**Table 1:** Prevalence of early initiation of breastfeeding (EIBF) by study factors among children aged 0–23 months in India, NFHS 2015–2016

	Total population (India)			Rural			Urban		
	N*	Prevalence (95% CI)	*P* value	N*	Prevalence (95% CI)	*P* value	N*	Prevalence (95% CI)	*P* value
EIBF									
Yes	39,048	41.5 (40.9–42.1)	< 0.001	27,951	41.0 (40.3–41.6)	< 0.001	11,097	42.9 (41.7–44.2)	< 0.001
No	55,056	58.5 (57.9–59.1)		40,309	59.1 (58.4–59.7)		14,747	57 (55.8–58.3)	
Socio-economic factors									
Maternal working status									
Not working	6,228	16.0 (15.3–16.7)	0.064	4,279	15.3 (14.6–16.1)	0.135	1,949	17.6 (16.1–19.2)	0.542
Working	772	2.0 (1.8–2.2)		597	2.1 (1.9–2.4)		175	1.6 (1.2–2.0)	
Maternal education									
No education	9,344	23.9 (23.2–24.6)	< 0.001	7,919	28.3 (27.5–29.2)	< 0.001	1,425	12.8 (11.7, 14.1)	< 0.001
Primary	5,125	13.1 (12.6–13.7)		3,980	14.2 (13.7–14.8)		1,145	10.3 (9.1–11.7)	
Secondary and above	24,578	62.9 (62.1–63.8)		16,052	57.4 (56.5–58.4)		8,526	76.8 (75.1–78.5)	
Father’s education									
No education	1,122	2.9 (2.6–3.1)	0.0186	888	3.2 (2.9–3.5)	0.020	234	2.1 (1.6–2.7)	0.149
Primary	4,879	12.5 (11.9–13.1)		3,446	12.3 (11.7–13.0)		1,433	12.9 (11.7–14.3)	
Secondary and above	972	2.5 (2.2–2.8)		522	1.9 (1.7–2.1)		450	4.1 (3.4–4.9)	
Household wealth index									
Poor	17,804	45.6 (44.6–46.6)	< 0.001	16,226	58.1 (57.1–59.1)	< 0.001	1,578	14.2 (12.9–15.7)	0.030
Middle	8,226	21.1 (20.4–21.8)		6,093	21.8 (21.1–22.5)		2,133	19.2 (17.8–20.8)	
Rich	13,018	33.3 (32.4–34.3)		5,632	20.2 (19.4–20.9)		7,386	66.6 (64.6–68.5)	
Individual factors									
Mother’s age									
15–19 years	2,302	5.9 (5.5–6.3)	< 0.001	1,834	6.6 (6.2–7.0)	< 0.001	468	4.2 (3.5–5.0)	0.037
20–34 years	34,837	89.2 (88.8–89.7)		24,683	88.3 (87.8–88.8)		10,154	91.5 (90.5–92.4)	
35–49 years	1,909	4.9 (4.6–5.2)		1,434	5.1 (4.8–5.5)		475	4.3 (3.7–5.0)	
Marital status									
Currently married	38,715	99.2 (99.0–99.3)	0.045	27,690	99.1 (98.9–99.2)	0.036	11,025	99.4 (99.1–99.6)	0.474
Never married / formerly married (divorced/separated/widow)	333	0.8 (0.7–0.9)		261	0.9 (0.7–1.1)		71	0.6 (0.4–0.9)	
Health service factors									
Place of delivery									
Home	5,781	14.8 (14.2–15.4)	< 0.001	4,965	17.8 (17.0–18.5)	< 0.001	816	7.4 (6.4–8.5)	< 0.001
Health facility	33,267	85.2 (84.6–85.8)		22,986	82.2 (81.5–83.0)		10,281	92.7 (91.5–93.7)	
Mode of delivery									
Vaginal delivery	33,009	84.5 (83.9–85.2)	< 0.001	24,611	88.1 (87.4–88.6)	< 0.001	8,398	75.7 (73.9–77.4)	< 0.001
Caesarean delivery	6,039	15.5 (14.8–16.2)		3,340	12.0 (11.4–12.6)		2,698	24.3 (22.6–26.1)	
Type of delivery assistance									
Health professionals	28,891	74.0 (73.2–74.8)	< 0.001	19,629	70.2 (69.4–71.1)	< 0.001	9,262	83.5 (81.9–84.9)	< 0.001
Traditional birth attendants	3,242	8.3 (7.9–8.8)		2,771	9.9 (9.4–10.5)		472	4.3 (3.6–5.0)	
Other non-health professionals	6,669	17.1 (16.5–17.7)		5,343	19.1 (18.4–19.9)		1,326	12.0 (10.7–13.3)	
Antenatal clinic visits									
None	5,182	13.3 (12.7–13.9)	< 0.001	4,259	15.2 (14.6–16.0)	< 0.001	923	8.3 (7.3–9.5)	< 0.001
1–3	11,551	29.6 (28.9–30.3)		9,286	33.2 (32.4–34.1)		2,265	20.4 (18.9–22.0)	
Four or more	22,015	56.4 (55.5–57.3)		14,211	50.8 (49.8–51.9)		7,805	70.3 (68.5–72.0)	
Environmental factor									
Geographical region									
North	3,853	9.9 (9.3–10.4)	< 0.001	2,618	9.4 (8.8–9.9)	< 0.001	1,235	11.1 (10.0–12.4)	< 0.001
South	8,527	21.8 (20.8–22.9)		5,017	18.0 (16.9–19.0)		3,510	31.6 (29.7–33.6)	
East	10,321	26.4 (25.3–27.6)		8,503	30.4 (29.2–31.7)		1,818	16.4 (14.8–18.1)	
West	6,440	16.5 (15.4–17.7)		3,710	13.3 (12.3–14.4)		2,730	24.6 (22.3–27.1)	
Central	7,790	20.0 (19.2–20.7)		6,260	22.4 (21.5–23.4)		1,530	13.8 (12.8–14.9)	
North-East	2,117	5.4 (5.1–5.8)		1,843	6.6 (6.1–7.1)		274	2.5 (2.2–2.7)	

Prevalence (EIBF): The proportion of children 0–23 months of age who were put to the breast within one hour of birth; N*: the weighted total number varies between categories due to missing data

### Determinants of early initiation of breastfeeding in India

In the total population of India, mothers with secondary and above education were significantly more likely to timely initiate breastfeeding compared to mothers with no schooling [Adjusted Odds ratio (AOR): 1.40, 95% CI: 1.24–1.59; P < 0.001) **[**Table 2**]**. Conversely, higher partner education was associated with delayed EIBF (AOR: 0.75, 95% CI: 0.62–0.91; *P* = 0.004 for secondary and above education). Women who had been formerly married were more likely to initiate breastfeeding within 1 h of birth (AOR: 2.13, 95% CI: 1.31–3.46; *P* = 0.002). Caesarean birth was associated with delayed EIBF in the Indian population (AOR: 0.53, 95% CI: 0.49–0.56; *P* < 0.001). Mothers who were assisted by TBAs or other non-health professionals were less likely to practice EIBF compared to those who received assistance from health professionals (AOR: 0.87, 95% CI: 0.80–0.94; *P* < 0.001 and AOR: 0.84, 95% CI: 0.79–0.90; P < 0.001 respectively). Mothers who received four or more antenatal care visits and those who gave birth in a health facility were more likely to practice EIBF compared to those who received no ANC visits and those who birthed at home (AOR: 1.43, 95% CI: 1.33–1.55; *P* < 0.001 and AOR: 1.22, 95% CI: 1.14–1.31; *P* < 0001, respectively). Concerning geographic regions, mothers who resided in the North-East were more likely to initiate breastfeeding in the first-hour post-birth compared to those who resided in the North (AOR: 3.64, 95% CI: 2.89–4.60; *P* < 0.001). Residence in the Southern, Eastern and Western regions was significantly associated with EIBF practice in India **[**Table 2**]**.

**Table 2:** Determinants of early initiation of breastfeeding in the total population of Indian mothers, NFHS 2015–2016

	Unadjusted OR	95% CI	*P* value	Adjusted^a^ OR	95% CI	*P* value
Socioeconomic factors						
Maternal working status						
Not working	1.00			1.00		
Working	0.88	0.76–1.00	0.061	0.87	0.75–1.00	0.044
Maternal education						
No education	1.00			1.00		
Primary	1.19	1.11–1.27	< 0.001	1.09	0.93–1.26	0.285
Secondary and above	1.38	1.32–1.44	< 0.001	1.40	1.24–1.59	< 0.001
Father’s education						
No education	1.00			1.00		
Primary	1.12	1.00–1.27	0.058	0.95	0.83–1.08	0.449
Secondary and above	0.95	0.80–1.12	0.514	0.75	0.62–0.91	0.004
Individual factors						
Marital status						
Currently married	1.00			1.00		
Never married / formerly married (divorced/separated/widow)	1.22	1.00–1.48	0.046	2.08	1.30–3.35	0.002
Preceding birth interval						
No previous birth	1.00			1.00		
< 24 months	0.99	0.94–1.05	0.819	1.16	1.02–1.34	0.027
> 24 months	1.06	1.01–1.102	0.011	1.15	1.03–1.28	0.010
Health Service factors						
Mode of delivery						
Vaginal delivery	1.00			1.00		
Caesarean delivery	0.66	0.62–0.70	< 0.001	0.53	0.49–0.56	< 0.001
Type of delivery assistance						
Health professionals	1.00			1.00		
Traditional birth attendants	0.72	0.67–0.77	< 0.001	0.87	0.80–0.94	< 0.001
Other non-health professionals	0.74	0.70–0.78	< 0.001	0.84	0.79–0.90	< 0.001
Antenatal clinic visits						
None	1.00			1.00		
1–3	1.14	1.07–1.21	< 0.001	0.98	0.91–1.05	0.531
Four or more	1.65	1.55–1.76	< 0.001	1.43	1.33–1.55	< 0.001
Place of delivery						
Home	1.00			1.00		
Health facility	1.43	1.36–1.51	< 0.001	1.22	1.14–1.31	< 0.001
Environmental factor						
Geographical region						
North	1.00			1.00		
South	2.05	1.89–2.23	< 0.001	1.88	1.57–2.25	< 0.001
East	1.50	1.39–1.61	< 0.001	1.50	1.26–1.78	< 0.001
West	2.43	2.20–2.68	< 0.001	1.92	1.53–2.40	< 0.001
Central	0.85	0.80–0.91	< 0.001	0.87	0.75–1.01	0.070
North East	3.50	3.17–3.86	< 0.001	3.64	2.89–4.60	< 0.001

Statistically significant (using confidence interval and *P* < 0.05) study factors from multivariate models are shown; ^**a**^Adjusted for listening to radio/television, reading newspaper/magazine, religion, caste/tribe, religion, child age, baby sex, place of residence, birth order, maternal age and maternal body mass index

### Determinants of early initiation of breastfeeding in rural-urban India

Among the rural population, higher maternal education increased the odds of EIBF (AOR: 1.40, 95% CI: 1.23–1.59; P < 0.001 for secondary and above education) **[**Table 3**]**. Rural mothers who had previously been married were more likely to practice EIBF (AOR: 2.00, 95% CI: 1.17–3.41, *P* = 0.011). Urban mothers with husbands who had secondary and above education were also less likely to initiate breastfeeding compared to those who had no education (AOR: 0.59, 95% CI: 0.37–0.94; *P* = 0.028). The association between partner education and EIBF was attenuated in rural areas (AOR: 0.81, 95% CI: 0.66–1.00; *P* = 0.050).

**Table 3:** Determinants of early initiation of breastfeeding in urban and rural population of Indian mothers, NFHS 2015–2016

	Urban residence						Rural residence					
	Unadjusted OR	95% CI	*P* value	Adjusted^a^ OR	95% CI	P value	Unadjusted OR	95% CI	*P* value	Adjusted^a^ OR	95% CI	*P* value
Socio-economic factors												
Maternal working status												
Not working	1.00			1.00			1.00			1.00		
Working	0.88	0.62–1.24	0.471	0.88	0.62–1.24	0.460	0.88	0.76–1.02	0.099	0.87	0.75–1.01	0.071
Maternal education												
No education	1.00			1.00			1.00			1.00		
Primary	1.21	0.99–1.48	0.063	1.03	0.70–1.51	0.881	1.18	1.10–1.25	< 0.001	1.08	0.92–1.26	0.353
Secondary and above	1.34	1.17–1.53	< 0.001	1.33	0.95–1.87	0.093	1.38	1.32–1.45	< 0.001	1.40	1.23–1.59	< 0.001
Father’s education												
No education	1.00			1.00			1.00			1.00		
Primary	0.84	0.59–1.21	0.349	0.76	0.51–1.12	0.168	1.18	1.05–1.33	0.006	1.00	0.87–1.14	0.981
Secondary and above	0.66	0.45–0.98	0.041	0.59	0.37–0.94	0.028	1.02	0.85–1.23	0.798	0.81	0.66–1.00	0.050
Individual factors												
Marital status												
Currently married	1.00			1.00			1.00			1.00		
Never married / formerly married (divorced/separated/widow)	1.13	0.71–1.78	0.505	2.31	0.76–7.03	0.141	1.25	1.01–1.56	0.037	1.96	1.17–3.28	0.010
Health service factors												
Mode of delivery												
Vaginal delivery	1.00			1.00			1.00			1.00		
Caesarean delivery	0.57	0.51–0.64	< 0.001	0.51	0.44–0.57	< 0.001	0.69	0.64–0.73	< 0.001	0.54	0.50–0.58	< 0.001
Type of delivery assistance												
Health professionals	1.00			1.00			1.00			1.00		
Traditional birth attendants	0.67	0.55–0.83	< 0.001	0.83	0.66–1.05	0.118	0.73	0.68–0.79	< 0.001	0.88	0.81–0.96	0.002
Other non-health professionals	0.72	0.62–0.84	< 0.001	0.81	0.68–0.96	0.015	0.74	0.70–0.79	< 0.001	0.85	0.80–0.91	< 0.001
Antenatal Clinic visits												
None	1.00			1.00			1.00			1.00		
1–3	0.91	0.76–1.10	0.350	0.82	0.66–1.02	0.070	1.19	1.12–1.27	< 0.001	1.02	0.95–1.09	0.663
Four or more	1.32	1.11–1.57	0.002	1.24	1.00–1.53	0.051	1.74	1.63–1.86	< 0.001	1.48	1.36–1.60	< 0.001
Place of delivery												
Home	1.00			1.00			1.00			1.00		
Health facility	1.53	1.30–1.81	< 0.001	1.45	1.18–1.78	< 0.001	1.40	1.32–1.48	< 0.001	1.19	1.11–1.27	< 0.001
Environmental factors												
Geographical region												
North	1.00			1.00			1.00			1.00		
South	2.30	1.97–2.69	< 0.001	2.21	1.57–3.09	< 0.001	1.92	1.74–2.11	< 0.001	1.67	1.35–2.05	< 0.001
East	1.87	1.56–2.25	< 0.001	1.39	0.94–2.04	0.100	1.41	1.30–1.53	< 0.001	1.56	1.29–1.88	< 0.001
West	2.52	2.09–3.04	< 0.001	1.63	1.08–2.46	0.019	2.40	2.14–2.68	< 0.001	2.14	1.66–2.75	< 0.001
Central	0.77	0.67–0.89	< 0.001	0.67	0.49–0.91	0.011	0.87	0.80–0.93	< 0.001	0.95	0.80–1.13	0.556
North-East	2.99	2.48–3.60	< 0.001	2.46	1.60–3.79	< 0.001	3.52	3.15–3.94	< 0.001	4.04	3.09–5.27	< 0.001

Statistically significant (using confidence interval and P < 0.05) study factors from multivariate models are shown; ^**a**^Adjusted for listening to radio/television, reading newspaper/magazine, religion, caste/tribe, religion, child age, baby sex, maternal age, birth order, birth interval and maternal body mass index

Both rural and urban mothers who gave birth in health facilities had a higher likelihood of engaging in EIBF (AOR: 1.19, 95% CI: 1.11–1.27; *P* < 0.001 for rural and AOR: 1.45, 95% CI: 1.18–1.78; P < 0.001 for urban). Caesarean birth and delivery assistance from TBAs or other non-health professionals were associated with decreased odds of EIBF in rural areas (AOR: 0.54, 95% CI: 0.50–0.58; *P* < 0.001 for caesarean births; AOR: 0.88, 95% CI: 0.81–0.96; *P* = 0.002 for TBAs and AOR: 0.85, 95% CI: 0.80–0.91; P < 0.001 for other non-health professionals). In urban areas, caesarean birthing and receiving birthing assistance from non-health professionals, who were not TBAs, reduced the likelihood of initiating breastfeeding within 1 h of birth compared to vaginal birthing and receiving assistance from health professionals (AOR: 0.51, 95% CI: 0.44–0.57; *P* < 0.001 for caesarean births and AOR: 0.81, 95% CI: 0.68–0.96; *P* = 0.01 for other non-health professionals). Rural mothers who attended 4 or more ANC visits were more likely to timely initiate breastfeeding (AOR: 1.48, 95% CI: 1.36–1.60; *P* < 0.001).

Among rural mothers, the likelihood of EIBF was increased in those who resided in the North-Eastern, Southern, Western and Eastern regions, similar to the results for the total population and urban residence **[**Table 3**]**. In urban areas, mothers in the North-Eastern, Western and Southern regions were more likely to breastfeed within the first hour post-birth compared to those in the Northern region (AOR: 2.46, 95% CI: 1.60–3.79; P < 0.001 for North-East; AOR: 1.63, 95% CI: 1.08–2.46; P < 0.001 for West and AOR: 2.21, 95% CI: 1.57–3.09; P < 0.001 for South). Central region mothers were 33% less likely to timely breastfeed their babies compared to North region mothers (AOR: 0.67, 95% CI: 0.49–0.91; *P* = 0.011) **[**Table 3**]**.

## Discussion

Our study showed that 41.5% of Indian mothers initiated breastfeeding within 1-h post-birth. This proportion was almost similar to mothers who resided in both rural (41.0%) and urban (42.9%) areas, with substantial difference among mothers who resided in urban areas compared to those who lived in rural areas of India. The prevalence and likelihood of EIBF was highest in Indian mothers who reported frequent health service contacts and those with higher educational attainment, with minor differences in both rural and urban dwellers. The prevalence of EIBF varied in the regions of India. Residence in the Southern, Eastern, Western and North-Eastern regions were associated with EIBF practice in India. This was despite the fact that the North-East region had the lowest percentage of EIBF in all populations. Residing in rural areas of the Central region was associated with delayed EIBF. The present study found that birthing at home, by caesarean delivery or receiving delivery assistance from non-health professionals were associated with decreased likelihood of EIBF in all populations.

The present study showed that India’s EIBF prevalence was well below the recommended level (41.5% as against the expected 90%). Nevertheless, it is important to note that India’s current EIBF rate is due to the implementation of an array of national child health programmes (i.e., the National Health Mission in partnership with the Indian Academy of Paediatrics and mass media campaigns) [15] that saw EIBF prevalence  increase from 24.5% in 2006 [23] to 41.5% in 2016. This improvement in EIBF prevalence demonstrates not only that infant feeding interventions that are context-specific could improve breastfeeding practices, but that well-coordinated child health programmes could make a significant impact in children’s health and well-being in both the short- and long-term [8, 41].

Consistent with findings from a systematic review conducted for South Asia [42] and studies from India [43], Pakistan [44], Bangladesh [45] and Nepal [46], our study indicated that higher maternal educational attainment in the total population was associated with EIBF compared to those with no schooling. Evidence has shown that higher maternal education has significant impact on child nutrition and well-being [47–50]. This may be due to the increased receptivity of a mother with formal education to health promotion campaigns and their empowerment status within the household to make informed health-related decisions [51, 52]. The association between higher maternal educational attainment and EIBF highlights the wide-ranging importance of improving women’s access to quality education, in line with the Sustainable Development Goal–4 (SDG–4), which aims to ensure that all girls and boys complete free, equitable and quality primary and secondary education by the year 2030 [53].

Difficulties in accessing health care services such as ANC have been shown to form significant barriers in initiating breastfeeding within the first hour of birth [54–56]. Our study indicated that receiving four or more ANC sessions was associated with increased likelihood of EIBF in the total population. This finding is consistent with previous studies conducted in Sri Lanka [19, 57], Nepal [46] and Bangladesh [58] which found that no or less than four ANC visits was associated with delayed EIBF. These findings suggest that the health messages provided during ANC sessions could improve mothers’ adherence to the WHO breastfeeding recommendations [59]. For example, step 3 of the revised Baby-Friendly Hospital Initiative (BFHI) indicates that all pregnant women and their families should be informed of the importance and management of breastfeeding in ANC sessions [60]. Increasing ANC uptake would ensure improvements in India’s BFHI, a global breastfeeding strategy to promote, protect and support optimal lactation among new mothers [61]. Between 2015 and 2018, the World Breastfeeding Trends Initiative – India (WBTi) scored the country zero out of 10 indicators used to assess the country’s BFHI as there was no data on the total hospitals (both public & private) and maternity facilities designated or reassessed as “Baby Friendly” in line with global criteria [62, 63]. This implies that efforts need to be made to realign or refine current breastfeeding programs (such as the Mother’s Absolute Affection programme [64]) in line with global best practices.

Since the year 1997, improving ANC access has been a priority for the Government of India, where key strategic initiatives have resulted in free-of-charge maternal and child health services, as well as the 24-h operation of primary health care centres in the rural areas [65–67]. Despite these interventions in health service delivery, the utilisation of these services has been limited [24]. This is evident in the current study, with demographic data showing that 70.3% of urban mothers who initiated breastfeeding within 1-h of birth attended four or more ANC visits compared to 50.8% of rural mothers, further highlighting the disparities in health service utilisation in India. This finding is in line with previous studies which demonstrated wide rural-urban disparities in ANC utilisation in India [68, 69]. The continued improvement in public health infrastructure in conjunction with the development of health programmes that aim to increase ANC utilisation will likely have a significant impact on EIBF and subsequent infant health in India.

The present study showed differences in rural-urban residence in relation to the increased likelihood of EIBF. Higher maternal education and frequent ANC (≥4) visits were associated with EIBF among mothers residing in rural areas compared to those living in the urban areas. It is uncertain as to why mothers in rural compared to urban areas would be more inclined to timely initiate breastfeeding; however, government funded rural-specific MCH interventions such as the NRHM – which aimed to address the health needs of under-served rural areas – may have played a role [21]. To our knowledge, this is the first study to consider the determining factors of EIBF in rural-urban settings in India. Therefore, further studies that explore the potential reasons for why rural mothers may be more likely to timely initiate breastfeeding compared to their urban counterparts are warranted.

Appropriate birthing-related circumstances are essential in encouraging mothers to timely initiate breastfeeding [42]. Our analysis showed that birthing in a health facility was associated with EIBF compared to home birthing, regardless of rural-urban residence. These results are consistent with the literature from Bangladesh [58], Sri Lanka [70] and Nepal [46]. In addition, delivery assisted by health professionals was associated with EIBF compared to TBAs or other non-health professionals’ assisted delivery. Notably, the mode of delivery was also of great significance, with caesarean birthing potentially negating some of the positive effects of health facility birthing on EIBF practice. Consistent with previous Indian studies, as well as the broader literature [43, 71–73], we found that caesarean birth was associated with delayed EIBF compared to vaginal birth. A recent systematic review has suggested that the impact of caesarean birth on EIBF may be due to the post-operative care that possibly disrupts the early skin-to-skin contact which supports EIBF [74]. Despite this challenge, evidence suggests that EIBF is feasible even after caesarean delivery if health professionals are well-trained to provide the necessary support and guidance to the mother [75, 76]. It is, therefore, essential that initiatives aimed at increasing breastfeeding should include the training of health professionals and establishment of ‘Baby Friendly’ health facilities to appropriately support mothers to breastfeed within the first hour of birth [77].

The present study found that the odds of timely initiation of breastfeeding were highest in the North-East, South, East and West of India compared to the Northern region, irrespective of rural-urban residence. In contrast, the Central region was associated with delayed EIBF in both the total population and urban residence compared to the Northern region, which is consistent with previous studies conducted in India that found reduced odds of EIBF in the Central region of India [19, 43].The underlying reasons for the regional variations in EIBF practice have not yet been adequately elucidated in the literature. However, plausible reasons may be due to the local cultural attitudes such as the negative perceptions towards the use of the first milk (colostrum) [78], advice from mothers-in-law that does not promote optimal breastfeeding [42] and the mother’s prenatal intention not to breastfeed [79]. In India, there is a need to provide region-specific policies and interventions that target mothers in their local communities, as well as the involvement of family members and community leaders in order to improve EIBF practice. Our analysis also indicated that higher father’s education and being formerly or never married never were associated with delayed EIBF. Additional studies are needed to investigate the factors associated with regional variations of EIBF, as well as the impact of father’s education status and marital status on EIBF in India.

### Study limitations and strengths

The study has methodological limitations that should be considered. First, we used cross-section data for the study, indicating that a clear temporal association between the study factors and EIBF cannot be established. Second, the information on the study factors and outcome variable were based on self-reporting. This is a source of recall or measurement bias, which could result in an overestimation or underestimation of the association between the study factors and EIBF. Third, a lack of assessment of unmeasured confounding factors (instrumental vaginal birth, cultural reasons, health professional’s knowledge of EIBF or family dynamics) may have affected the association between the primary and secondary outcomes.

Despite the limitations, the study has strengths. First, we believe that the possible effect of selection bias is unlikely to impact the study findings based on the nationally representative nature of the sample size and the high response rates (94–99.6%). Second, the NFHS-4 data, including the study factors and EIBF were collected by trained personnel who used standardised questionnaires to ensure consistency across all Indian states and territories. Finally, our study provides relevant contextual evidence on key modifiable determinants of EIBF in one of the world’s largest populations to advocate for appropriate policies and interventions that seek to promote EIBF practice in India.

## Conclusion

Our study suggests that less than half of Indian mothers initiated breastfeeding within 1-h post-birth (41.5%), with a significant difference in both rural and urban EIBF prevalence (41.0 and 42.9%, respectively). Higher maternal education, frequent ANC visits, birthing in a health facility and residence in the North-East, South, West and East regions were associated with EIBF in India, regardless of rural-urban residence. In contrast, we found that mothers delayed breastfeeding after birth if they lived in the Central region, received delivery assistance from non-health professionals or gave birth through caesarean section. In India, it is essential that health promotion campaigns to improve EIBF should be region-specific and should focus on mothers with no schooling and those with limited access to healthcare facilities to maximise impacts.

## Additional file

Additional file 1: Characteristics of the study population, 2015–2016 India NFHS. (DOCX 24 kb)

## Data Availability

The analysis was based on the datasets collected for the India Demographic and Health Survey. Information on the data and content can be assessed at http://www.dhsprogram.com/data/available-datasets.cfm.

## Abbreviations

BFHI: Baby-Friendly Hospital Initiative
DHS: Demographic and health survey
EIBF: Early initiation of breastfeeding
IIPS: International Institute for Population Sciences
NFHS: National Family and Health Survey
NRHM: National Rural Health Mission
OR: Odds ratio
UNICEF: United Nations Children’s Fund
WHO: World Health Organization
