# Supplementary material for: National and rural-urban prevalence and determinants of early initiation of breastfeeding in India
Source: BMC Public Health. 2019 Jul 8;19:896. doi: 10.1186/s12889-019-7246-7 (PMC6615079; doi:10.1186/s12889-019-7246-7)
Supplement: Supplementary file 1 — Characteristics of the study population, 2015–2016 India NFHS. (DOCX 24 kb) [file 12889_2019_7246_MOESM1_ESM.docx]

**Additional file 1: Characteristics of the study population, 2015–2016 India NFHS**

|  | **India Population**  **(N=94,104)** | | **Rural (N=68,260)** | | **Urban (N=25,843)** | |
| --- | --- | --- | --- | --- | --- | --- |
|  | **N*** | **% (95%CI)** | **N*** | **% (95%CI)** | **N*** | **% (95%CI)** |
| ***Socio-economic factors*** |  |  |  |  |  |  |
| **Maternal working status** |  |  |  |  |  |  |
| Not working | 14,566 | 15.5 (15.0-16.0) | 10,163 | 14.9 (14.4-15.4) | 4,403 | 17.0 (15.9-18.2) |
| Working | 1,952 | 2.1 (1.9-2.2) | 1,527 | 2.2 (2.1-2.4) | 425 | 1.6 (1.4-1.9) |
| **Maternal education** |  |  |  |  |  |  |
| No education | 25,677 | 27.3 (26.8-27.8) | 21,849 | 32.0 (31.4-32.6) | 3,828 | 14.8 (13.9-15.8) |
| Primary | 12,672 | 13.5 (13.2-13.8) | 9,931 | 14.6 (14.2-14.9) | 2,742 | 10.6 (9.9-11.3) |
| Secondary and above | 55,754 | 59.3 (58.7-59.8) | 36,481 | 53.4 (52.8-54.1) | 19,274 | 74.6 (73.4-75.8) |
| **Father's education** |  |  |  |  |  |  |
| No education | 2,763 | 2.9 (2.8-3.1) | 2,287 | 3.4 (3.1-3.6) | 476 | 1.8 (1.6-2.2) |
| Primary | 11,228 | 11.9 (11.5-12.4) | 8,037 | 11.8 (11.4-12.2) | 3,190 | 12.3 (11.4-13.4) |
| Secondary and above | 2,475 | 2.6 (2.5-2.8) | 1,325 | 1.9 (1.8-2.1) | 1,151 | 4.5 (4.0-5.0) |
| **Household wealth index** |  |  |  |  |  |  |
| Poor | 44,012 | 46.8 (46.1-47.4) | 40,561 | 59.4 (58.7-60.1) | 3,450 | 13.4 (12.4-14.3) |
| Middle | 19,052 | 20.3 (19.8-20.7) | 14,281 | 20.9 (20.4-21.4) | 4,772 | 18.5 (17.5-19.4) |
| Rich | 31,040 | 33.0 (32.3-33.6) | 13,418 | 19.7 (19.1-20.2) | 17,621 | 68.2 (66.8-69.5) |
| ***Individual factors*** |  |  |  |  |  |  |
| **Mother's age** |  |  |  |  |  |  |
| 15-19 years | 5,415 | 5.8 (5.5-6.0) | 4,389 | 6.4 (6.2-6.7) | 1,027 | 4.0 (3.5-4.5) |
| 20-34 years | 83,531 | 88.8 (88.5-89.1) | 60,008 | 87.9 (87.6-88.2) | 23,524 | 91.0 (90.3-91.7) |
| 35-49 years | 5,157 | 5.5 (5.3-5.7) | 3,864 | 5.7 (5.4-5.9) | 1,293 | 5.0 (4.5-5.6) |
| **Marital status** |  |  |  |  |  |  |
| Currently married | 93,386 | 99.2 (99.2-99.3) | 67,699 | 99.2 (99.1-99.3) | 25,687 | 99.4 (99.2-99.5) |
| Never married/formerly married (divorced/separated/widow) | 718 | 0.7 (0.6-0.7) | 562 | 0.8 (0.7-0.9) | 156 | 0.6 (0.5-0.7) |
| ***Health service factors*** |  |  |  |  |  |  |
| **Place of delivery** |  |  |  |  |  |  |
| Home | 16,750 | 17.8 (17.4-18.3) | 14,334 | 21.0 (20.4-21.6) | 2,416 | 9.3 (8.6-10.2) |
| Health facility | 77,354 | 82.2 (81.7-82.7) | 53,926 | 79.0 (78.4-79.6) | 23,428 | 90.7 (89.8-91.4) |
| **Mode of delivery** |  |  |  |  |  |  |
| Vaginal delivery | 76,067 | 80.8 (80.4-81.3) | 58,255 | 85.3 (84.9-85.8) | 17,811 | 68.9 (67.7-70.1) |
| Caesarean delivery | 18,037 | 19.2 (18.7-19.6) | 10,005 | 14.7 (14.2-15.1) | 8,032 | 31.1 (29.9-32.3) |
| **Type of delivery assistance** |  |  |  |  |  |  |
| Health professionals | 66,083 | 70.2 (69.7-70.8) | 45,270 | 66.3 (65.7-67.0) | 20,813 | 80.5 (79.4-81.6) |
| Traditional birth attendants | 9,071 | 9.6 (9.3-10.0) | 7,724 | 11.3 (10.9-11.7) | 1,347 | 5.2 (4.7-5.8) |
| Other non-health professionals | 18,339 | 19.5 (19.0-20.0) | 14,727 | 21.6 (21.0-22.1) | 3,612 | 14.0 (13.0-15.0) |
| **Antenatal clinic visits** |  |  |  |  |  |  |
| None | 15,073 | 16.0 (15.6-16.5) | 12,700 | 18.6 (18.1-19.2) | 2,373 | 9.2 (8.5-10.0) |
| 1-3 | 30,914 | 32.9 (32.4-33.4) | 24,758 | 36.3 (35.7-36.9) | 6,156 | 23.8 (22.7-24.9) |
| Four or more | 47,461 | 50.4 (49.8-51.0) | 30,368 | 44.5 (43.7-45.3) | 17,093 | 66.1 (64.9-67.4) |
| ***Environmental factor*** |  |  |  |  |  |  |
| **Geographical region** |  |  |  |  |  |  |
| North | 11,630 | 12.4 (12.0-12.7) | 7,752 | 11.4 (10.8-11.9) | 3,877 | 15.0 (14.1-16.0) |
| South | 16,920 | 18.0 (17.5-18.5) | 10,142 | 14.9 (14.1-15.6) | 6,778 | 26.2 (25.0-27.6) |
| East | 24,210 | 25.7 (25.2-26.3) | 20,314 | 29.8 (28.7-30.8) | 3,896 | 15.1 (14.1-16.1) |
| West | 11,793 | 12.5 (11.9-13.1) | 6,746 | 9.9 (9.2-10.6) | 5,047 | 19.5 (17.9-21.3) |
| Central | 26,213 | 27.9 (27.4-28.3) | 20,438 | 29.9 (29.1-30.9) | 5,775 | 22.4 (21.4-23.3) |
| North-East | 3,339 | 3.5 (3.4-3.7) | 2,869 | 4.2 (3.9-4.5) | 470 | 1.8 (1.7-2.0) |

N*: the weighted total number varies between categories due to missing data
